# Protective effects of levamisole, acetylsalicylic acid, and α-tocopherol against dioxin toxicity measured as the expression of AhR and COX-2 in a chicken embryo model

**DOI:** 10.1007/s00418-016-1528-2

**Published:** 2016-12-10

**Authors:** Kinga Gostomska-Pampuch, Alicja Ostrowska, Piotr Kuropka, Maciej Dobrzyński, Piotr Ziółkowski, Artur Kowalczyk, Ewa Łukaszewicz, Andrzej Gamian, Ireneusz Całkosiński

**Affiliations:** 1 Department of Immunology of Infectious Diseases, Institute of Immunology and Experimental Therapy, Polish Academy of Sciences, Weigla 12, 53-114 Wrocław, Poland; 2 Laboratory of Neurotoxicology and Environmental Diagnostics, Wroclaw Medical University, Bartla 5, 51-618 Wrocław, Poland; 3 Department of Animal Physiology and Biostructure, Wroclaw University of Environmental and Life Sciences, Norwida 31, 50-375 Wrocław, Poland; 4 Department of Conservative Dentistry and Pedodontics, Wroclaw Medical University, Krakowska 26, 50-425 Wrocław, Poland; 5 Department of Pathomorphology, Wroclaw Medical University, Marcinkowskiego 1, 50-368 Wrocław, Poland; 6 Institute of Animal Breeding, Wroclaw University of Environmental and Life Sciences, Chełmońskiego 38c, 51-630 Wrocław, Poland; 7 Department of Medical Biochemistry, Wroclaw Medical University, Chałubińskiego 10, 50-368 Wrocław, Poland; 8 Wroclaw Research Centre EIT+, Wrocław, Poland

**Keywords:** TCDD, AhR, COX-2, Chicken embryo, Histopathology

## Abstract

Polychlorinated dibenzo-*p*-dioxins and dibenzofurans (dioxins) are classed as persistent organic pollutants and have adverse effects on multiple functions within the body. Dioxins are known carcinogens, immunotoxins, and teratogens. Dioxins are transformed in vivo, and interactions between the products and the aryl hydrocarbon receptor (AhR) lead to the formation of proinflammatory and toxic metabolites. The aim of this study was to determine whether α-tocopherol (vitamin E), acetylsalicylic acid (ASA), and levamisole can decrease the amount of damage caused by dioxins. Fertile Hubbard Flex commercial line chicken eggs were injected with solutions containing 2,3,7,8-tetrachlorodibenzo-*p*-dioxin (TCDD) or containing TCDD and the test compounds. The chicken embryos and organs were analyzed after 7 and 13 days. The levels at which AhR and cyclooxygenase-2 (COX-2) proteins (which are induced during inflammation) were expressed were evaluated by performing immunohistochemical analyses on embryos treated with TCDD alone or with TCDD and the test compounds. TCDD caused developmental disorders and increased AhR and COX-2 expression in the chicken embryo tissues. Vitamin E, levamisole, ASA, and ASA plus vitamin E inhibited AhR and COX-2 expression in embryos after 7 days and decreased AhR and COX-2 expression in embryos after 13 days. ASA, levamisole, and ASA plus vitamin E weakened the immune response and prevented multiple organ changes. Vitamin E was not fully protective against developmental changes in the embryos.

## Introduction

Polychlorinated dibenzo-*p*-dioxins and dibenzofurans (dioxins) are hazardous chemicals that are classed as persistent organic pollutants. Dioxins are persistent toxins, with half-lives of 7–8 years in humans. Dioxins are resistant to degradation in the environment, can be transported long distances in the air, and directly threaten environmental and human health (Całkosiński et al. 2011; Wrbitzky et al. 2001). Dioxins containing between four and six chlorine atoms per molecule, such as 2,3,7,8-tetrachlorodibenzo-*p*-dioxin (TCDD), are some of the most toxic man-made chemicals (Goldstone and Stegeman 2006; Stec et al. 2012).

Humans can be exposed to dioxins through skin contact (typically 2% of total exposure), inhaling air (typically 8% of total exposure), and ingesting contaminated water or food (typically 90% of total exposure) (Całkosiński et al. 2011). Inhaled dioxins mostly enter the body adsorbed onto particles of smoke and dust that become phagocytized by pneumocytes. The penetration of dioxins through the skin is facilitated by the lipid layer of the skin coming into direct contact with soot, ash, or contaminated clothing (Całkosiński et al. 2005). Dioxins are lipophilic, so accumulate in fat within biota. Humans are primarily exposed to dioxins through ingesting food containing dioxins (Travis and Nixon 1991). Dioxins mainly accumulate in the liver and in adipose tissue. In experiments using laboratory animals, dioxins have also been found to accumulate in the skin and muscles (Żukiewicz-Sobczak et al. 2012).

Dioxins cause a range of toxic effects in different species by activating the aryl hydrocarbon receptor (AhR). In the absence of a suitable ligand, the AhRs are found in the cytoplasm complexed with chaperones. Once a dioxin molecule binds to an AhR–chaperone complex the complex undergoes a conformational change and is transported to the nucleus. There, the AhR–chaperone complex dissociates, the dioxin molecule binds to the AhR, and the ligand–receptor complex forms a heterodimer with the AhR nuclear translocator protein. The heterodimer then binds to the xenobiotic response element (also called the dioxin response element), which is a specific enhancer sequence on a strand of DNA. The xenobiotic response element/dioxin response element is in the promoter region of the cytochrome P-450 CYP1A1 gene (Mimura and Fujii-Kuriyama 2003; Niemira et al. 2009; Walker et al. 1997). Activation of the promoter causes the transcription of genes responsible for metabolizing drugs and xenobiotics and ultimately causes metabolic changes and increased enzymatic activation of carcinogens (Struciński et al. 2011). The dioxin response element regulatory sequence is also present in other genes induced by the AhR, called the AhR gene battery (Niemira et al. 2009; Williams et al. 2005). It is believed that the physiological activator of the AhR induces fast on/off switching of signal transduction but that dioxin-induced toxicity is caused by the AhR being continually activated, disturbing homeostasis. In the absence of dioxins, the AhR plays roles in regulating the cell cycle and suppressing tumors (Marlowe and Puga 2005) and in controlling cell proliferation and differentiation (Akahoshi et al. 2006; Quintana et al. 2008; Tijet et al. 2006; Walisser et al. 2005). It has been found that TCDD causes a wide range of biochemical and toxicological effects, including teratogenicity and immunosuppression. TCDD also affects the expression of genes that control the synthesis and metabolism of enzymes, hormones, and growth factors. Dioxins therefore ultimately affect the reproductive, nervous, immune, and endocrine systems (Struciński et al. 2011).

It has recently been found that dioxins have proinflammatory and multidirectional effects that stem from free radicals being produced when dioxins undergo epoxidation, dechlorination, and hydroxylation reactions and from the stimulation of cyclooxygenase-2 (COX-2) (Rosińczuk and Całkosiński 2015). Lim et al. (2007) found that TCDD induces oxidative stress related to the generation of reactive oxygen species in various organs but decreases the concentrations of antioxidant enzymes, such as catalase, superoxide dismutase, glutathione reductase, and glutathione peroxidase. Hassoun et al. (2004) found that TCDD causes the production of superoxide anions, the peroxidation of lipids, and damage to DNA in liver and brain cells. Elevated levels of proinflammatory cytokines such as interleukin-1, interleukin-6, and particularly tumor necrosis factor have been found in animals treated with dioxins (Całkosiński 2008). These molecules stimulate COX-2 activity, leading to the biosynthesis of proinflammatory prostaglandins and thromboxanes (Hla and Neilson 1992). Prostaglandins can modulate cell adhesion, immune response, mitogenesis, cell proliferation, apoptosis, and angiogenesis processes. Increased COX-2 activity, increasing prostaglandin generation, is therefore involved in maintaining the inflammatory process and intensifying carcinogenesis (Majka et al. 2009). Teraoka et al. (2009) found that increased prostaglandin concentrations cause circulatory failure to occur in the brains of developing zebrafish, causing functional disorders and destructive changes such as apoptosis and increased albumin permeability in the mesencephalic vein.

In the study described here, we examined the negative effects of TCDD on embryogenesis in a chick embryo model. The model is objective because the effects of external factors and manipulations that can affect the results of an experiment are limited. Moreover, injecting test compounds once into the yolk (which is used by the embryo to provide energy) ensures that the test compounds are fully absorbed by the developing embryo. This makes it possible to determine the effects of different agents on the developing tissues and organs in different stages of embryogenesis. It has previously been found that the exposure of an embryo to TCDD affects hatchability and causes early or late embryonic death (Blankenship et al. 2003). Head et al. (2008) analyzed experimental data produced by a number of researchers and found an LD50 of 0.18 μg/kg for chickens, indicating that chickens are very sensitive to dioxins. Exposing bird embryos to dioxins causes structural and functional defects in the developing heart. It has been found that these defects include fewer coronary arteries forming, decreased luminal surface areas of the developing coronary arteries, and pathological leakages from the vascular system. Dioxins inhibit the growth of cardiomyocytes and affect the abilities of ventricular walls to proliferate (Ivnitski-Steele et al. 2004; Walker and Catron 2000). Blankenship et al. (2003) found that exposure to TCDD causes developmental abnormalities including edema, liver necrosis, and deformations of the eyes and extremities of the body.

Prolonged exposure to dioxins, the accumulation of dioxins in adipose tissue, and the slow elimination of dioxins from the body make the negative effects of dioxins very persistent. It is therefore important that substances are found that can protect against the negative effects of dioxins. The proinflammatory effects of TCDD can be eliminated using antioxidant compounds, such as α-tocopherol (vitamin E), or nonsteroidal anti-inflammatory drugs such as acetylsalicylic acid (ASA) (Całkosiński et al. 2014). It has previously been found that these pharmaceuticals are AhR antagonists that can inhibit the inflammatory reactions induced by dioxins (Kloser et al. 2011; MacDonald et al. 2004). Interest in levamisole and its immunomodulatory capacity has recently increased. Tests on cows and pigs have shown that levamisole can affect both primary and secondary immune responses and can restore the proper functions of effector cells during an immune response (Obmińska-Domoradzka and Całkosiński 1994; Sajid et al. 2006). However, no information on the ability of levamisole to protect against the effects of exposure to dioxins is available.

## Materials and methods

Ethical approval was not required for this study according to the EU Directive of September 22, 2010, on the protection of animals used for scientific purposes (2010/63/UE) and the Polish Act of January 15, 2015, on the protection of animals used for scientific or educational purposes (Polish Journal of Laws of 2015, item 266). All of the procedures involving animals were performed in accordance with the ethical standards of the institution or practice at which the studies were conducted.

### Chemicals

A standard solution containing 1 µg/ml TCDD (DD-2378-S; Greyhound Chromatography and Allied Chemicals, Birkenhead, UK) in 1% dimethyl sulfoxide (DMSO) was prepared. A solution of 500 mg of ASA (aspirin) (Hasco-Lek, Wrocław, Poland) in 833 μl of 1% DMSO was prepared. A 1 ml aliquot of a solution containing vitamin E as α-tocopherol acetate (Hasco-Lek) was mixed with 800 μl of 1% DMSO to give a final α-tocopherol acetate concentration of 300 mg/ml. An aqueous 10% levamisole (Vetoquinol Biowet, Gorzów Wielkopolski, Poland) solution was prepared. An aqueous 1% DMSO solution was used in the experiments. A 4% formalin solution buffered with phosphate buffer at pH 7.0 was used. Serotec rabbit anti-human AhR antibodies (AHP1107; Bio-Rad Laboratories, Hercules, CA, USA) and rabbit anti-mouse COX-2 antibodies (160126; Cayman Chemical Company, Ann Arbor, MI, USA) were used in the experiments.

### Experimental groups

Hubbard Flex line chicken eggs with a mean weight of 60 g were divided into seven groups. Each egg in six of the groups was injected with a test solution before being incubated. The groups and treatments are described below.

1. Control group—not injected—58 eggs.
2. Control Group 2—injected with 5 μl of 1% DMSO—60 eggs.
3. Injected with 5 μl of the TCDD standard solution (5 ng/egg, equivalent to 0.08 ng/g)—60 eggs.
4. Injected with 5 μl of the TCDD standard solution and 10 μl of the vitamin E solution (1.8 mg/egg, equivalent to 30 µg/g)—60 eggs.
5. Injected with 5 μl of the TCDD standard solution and 1.5 μl of the levamisole solution (0.15 mg/egg, equivalent to 2.5 µg/g)—59 eggs.
6. Injected with 5 μl of the TCDD standard solution and 5 μl of the ASA solution (3 mg/egg, equivalent to 50 µg/g)—60 eggs.
7. Injected with 5 μl of the TCDD standard solution, 10 μl of the vitamin E solution, and 5 μl of the ASA solution—59 eggs.

The TCDD dose was determined after inspecting previously published data (Cohen-Barnhouse et al. 2011; Henshel et al. 1997). The vitamin E dose was determined from the results of studies by Całkosiński (2008) and Całkosiński et al. (2015), the ASA dose from the results of studies by Dobrzyński et al. (2014) and Rosińczuk and Całkosiński (2015), and the levamisole dose from the results of studies by Obmińska-Domoradzka and Całkosiński (1994) and Purzyc and Całkosiński (1998).

The surface of each egg shell was disinfected with 70% ethanol; then, a small hole was drilled at the blunt end of the egg. With the egg horizontally orientated, the specified solution was injected into the yolk by inserting the needle of a Hamilton syringe through the air chamber to about 3 cm deep. The needle was then withdrawn and the egg vertically orientated before the hole was sealed with melted paraffin (Blankenship et al. 2003). The eggs were placed in a C-82 incubator (Jartom, Gostyń, Poland) the day after being injected. The incubator was kept at 37.6 °C and 55% humidity, and the eggs were turned 90° every hour. The eggs were candled on the seventh and 13th days of incubation, and the viability of each embryo was assessed. Some of the eggs containing living embryos were broken on the seventh and 13th days of incubation to allow the embryos to be collected and subjected to immunohistochemical analyses. Whole embryos were collected on the seventh day, and each embryo collected on the 13th day was dissected and the heart, liver, brain, and eyes collected. Each tissue sample was fixed in 4% buffered formalin solution and stored at 4 °C.

### Immunohistochemical analysis

For analysis, a tissue sample (fixed in 4% formalin) was washed in running water and then cut into smaller pieces. The pieces were then dehydrated by placing them in a series of aqueous solutions containing increasing concentrations of ethanol (70, 80, 96, then 100%). The pieces were then treated with methyl benzoate and embedded in paraffin. Each paraffin block was then cut into 5-µm-thick sections using a rotary microtome (Slee, Mainz, Germany). Each section was then analyzed to determine the degree of AhR and COX-2 expression. The reagents used in the immunohistochemical analyses were supplied by Dako (Agilent Technologies, Wilmington, DE, USA). Each tissue section was incubated at 58 °C overnight; then, the paraffin was removed using xylene. The sample was then hydrated by placing it in a series of alcohol solutions and then washed with distilled water. The sample was then subjected to heat-induced epitope retrieval, cooled, and washed with distilled water. Endogenous peroxidase was neutralized using peroxidase block; then, the slide was washed with distilled water and incubated with protein block. Primary antibodies (50–100 µl of rabbit anti-human AhR antibodies diluted by a factor of 80 and rabbit anti-mouse COX-2 antibodies diluted by a factor of 300) were then applied to the sample, and the sample was incubated for 1 h at room temperature. The sample was then washed with phosphate-buffered saline, and secondary antibody anti-rabbit immunoglobulins, HRP conjugated, were applied before the sample was incubated for 30 min. The peroxidase activity was developed by incubating the sample with a mixture containing 3,3ʹ-diaminobenzidine (1 ml of 3,3ʹ-diaminobenzidine substrate buffer and one drop of 3,3ʹ-diaminobenzidine chromogen). The sample was then rinsed with distilled water and counterstained with Mayer’s hematoxylin (Chempur, Piekary Śląskie, Poland). The sample was then washed with tap water, dehydrated in a series of ethanol solutions (80, 96, then 100%), washed with xylene, and sealed in Canadian balm (Chempur, Piekary Śląskie, Poland).

## Results

### AhR expression and the state of embryo development

#### Seven-day-old chicken embryos

**Fig. 1:**
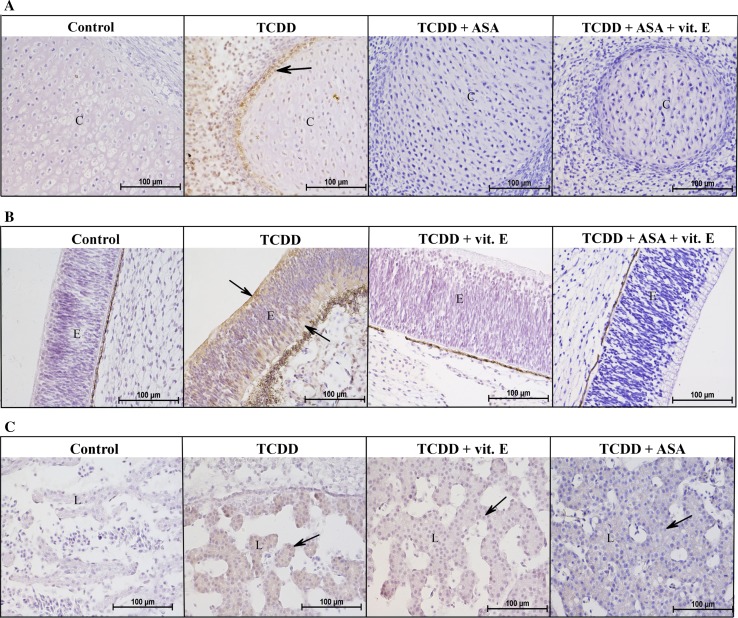
Comparison of Ah receptor expression in the selected organs and groups of 7-day-old chicken embryos. Control—group 1, TCDD—Group 3, TCDD + vit. E—Group 4, TCDD + ASA—Group 6, TCDD + ASA + vit. E—Group 7. **a** Cartilage (C) with perichondrium (magnification 400). Note the presence of the AhR in the TCDD group (*arrow*); **b** eye epithelium (E) (magnification ×400). Note the increased presence of the AhR in the TCDD group (*arrows*); **c** liver (L) (magnification ×400). Note the increased presence of the AhR in the groups with TCDD (*arrows*)

The AhR expression and developmental changes in the tissues from the chicken embryos collected on day 7 are summarized in Table 1 and shown in Fig. 1.

**Table 1:** Expression of Ah receptor and developmental changes in different tissues/organs and groups of 7-day-old chicken embryos

Organ\group	Control	DMSO	TCDD	Vit. E	LVM	ASA	ASA + vit. E
Epidermis							
Dorsal	+	+/−	+	+	−	−	−
Abdominal	−	−	++	−	−	−	−
Eye epithelium	−	−	++	−	−	−	−
Mesenchyme	+/−	−	+/−	+	−	−	−
Brain	−	−	+/−	+/−	−	−	−
Cartilage with perichondrium	−	−	+	−	−	−	−
Liver	−	−	+	−	−	−	−
Kidneys	−	−	+/−	−	−	+/−	+/−
Developmental disorders			Weaker ependyma cell proliferation in the brain		Weaker cell migration in the brain, underdeveloped ectodermal epithelium of the skin	Smaller chondrocytes	Delayed stage of development of the gray matter of the brain

−, lack of AhR expression; +/−, weak positive reaction, single cells; +, positive reaction; ++, strong positive reaction; Control, Group 1; DMSO, Group 2; TCDD, Group 3; vit. E, Group 4; LVM, Group 5; ASA, Group 6; ASA + vit. E, Group 7

The control group (Group 1) embryos collected on day 7 showed AhR expression, particularly in the mesenchyme and dorsal epidermis. The primordia of the spine, ribs, and limbs were well developed. The eye epithelium, the other auxiliary eye organs, and the brain were in the organization phase. The organs in the abdominal cavity were in the early stages of development and, like other organs, did not show positive reactions. The second control group (Group 2) embryos, treated with DMSO, showed weak positive reactions in the ridge epidermis, the lens and cornea ectodermal epithelia, and the apical zone of the differentiating olfactory epithelium.

Positive reactions were shown in most organs in the group injected with TCDD (Group 3). AhR expression was observed in the dorsal and abdominal epidermis and in the surface and internal parts of the eye epithelium (Fig. 1b). Weak positive reactions were found in the myoblasts and mesenchyme. A strong positive reaction was found on the outskirts of the liver, but a weaker reaction was found in the central part (Fig. 1c). Similarly, staining was more intense in the perichondrium than in cartilage (Fig. 1a). The gray matter of the brain lacked AhR expression, and the embryos were at an appropriate developmental stage compared with the control group. Weak proliferation and positive reactions were found in the ependyma cells in the brain. Nothing else indicated that embryo development had been delayed.

The embryos in the group injected with TCDD and vitamin E (Group 4) showed AhR expression in the myoblasts, mesenchymal and epidermal cells, and in the surfaces of the gray matter of the brain. Positive reactions and developmental disorders were not found in the parenchymal organs or nervous system.

The embryos in the group injected with TCDD and levamisole (Group 5) had weaker brain cell migration than in the control group. The eye epithelium was slightly thinner than in the control group but had a similar level of organization. The epidermis was only one cell thick but did not show a positive reaction. No AhR expression was observed in any organ.

The embryos in the group injected with TCDD and ASA (Group 6) had normal eye, epidermis, and brain development and lacked AhR expression. The kidneys and liver had slightly higher levels of organization than in the control group, especially the liver, in which the blood vessels had been almost completely closed by rapidly proliferating hepatocytes (Fig. 1c). Positive reactions were found in some liver and kidney samples.

The embryos in the group treated with TCDD, ASA, and vitamin E (Group 7) did not show positive reactions in the lens or eye epithelium (Fig. 1b). Development of the gray matter of the brain was delayed, but there was a highly developed area of migratory cells in the brain. The hypothalamus and medulla oblongata with the proliferative zone were further advanced than in the control group. The epidermis was also better developed than in the control group. No AhR expression was observed in any of these structures. Weak positive reactions were found in the proximal tubules of the kidneys.

#### Thirteen-day-old chicken embryos

**Fig. 2:**
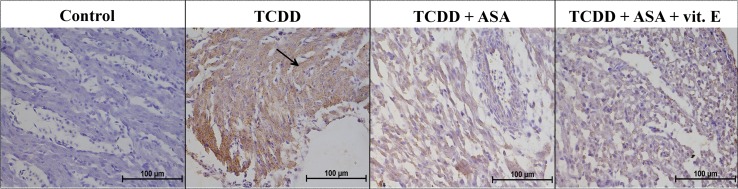
AhR expression in the hearts of the selected groups of 13-day-old chicken embryos (magnification ×400). Control—Group 1, TCDD—Group 3, TCDD + ASA—Group 6, TCDD + ASA + vit. E—Group 7. Note the hypertrophied cardiomyocytes with elevated AhR expression in TCDD group (*arrow*)

**Fig. 3:**
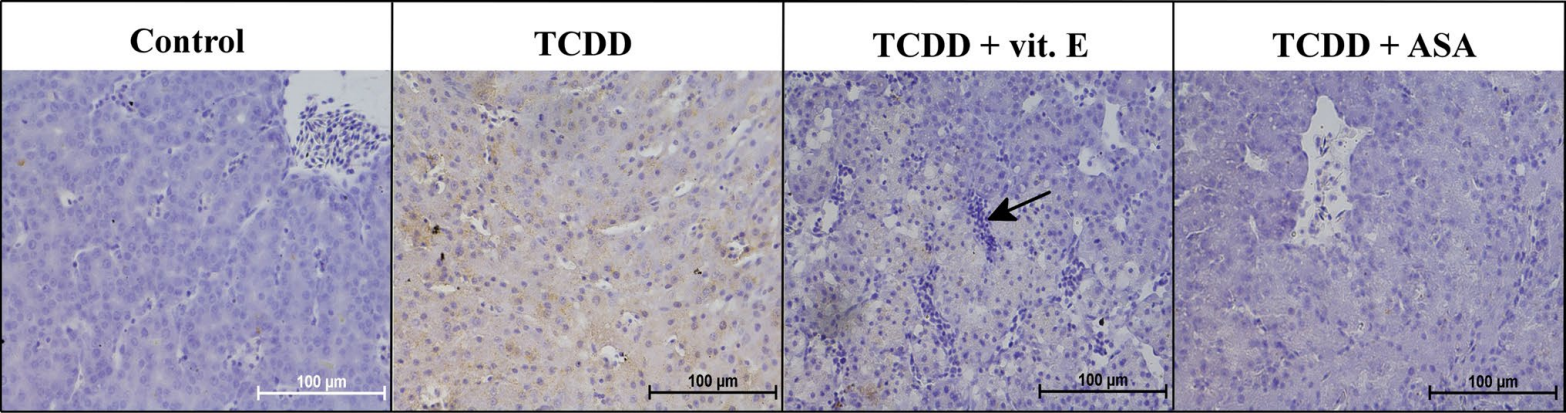
AhR expression in the livers of the selected groups of 13-day-old chicken embryos (magnification ×400). Control—Group 1, TCDD—Group 3, TCDD + vit. E—Group 4, TCDD + ASA—Group 6. Note the increased level of AhR expression in TCDD groups. In Group 4, degradation of hepatocytes with mild lymphocyte infiltration (*arrow*) can be observed

The AhR expression and developmental changes found in the chicken embryos collected on day 13 are presented in Table 2. The changes exposure to TCDD caused in the heart and liver are shown in Figs. 2 and 3, respectively.

**Table 2:** Expression of Ah receptor and developmental changes in different tissues/organs and groups of 13-day-old chicken embryos

Organ\group	Control	DMSO	TCDD	vit. E	LVM	ASA	ASA + vit. E
Heart							
Ventricle	−	+/−	++	++	+	+	+
Atrium	+/−	+/−	++	++	+	−	+/−
Apex	−	−	+/−	−	+	−	−
Developmental disorders		Myocyte hypertrophy	Myocyte hypertrophy	Excessive fat tissue			Hypoplasia
Liver							
Reaction	+/−	+/−	+	+/−	+/−	+/−	+/−
Developmental disorders		Hypoplasia	Hypoplasia, single steatosis	Steatosis, lymphocytic infiltrations, areas of necrosis		Single steatosis	Single steatosis
Brain							
Gray matter	−	−	+/−	+/−	+/−	−	−
White matter	+/−	+/−	+	+/−	+	+/−	+/−
Ependyma	+/−	+/−	+	−	−	−	−
Developmental disorders			Less developed outer layer of gray matter	Less developed outer layer of gray matter		Less developed outer layer of gray matter	Edema
Eye							
Reaction	−	+/−	+/−	−	−	+/−	+/−
Developmental disorders			Developmental disorder of eye epithelium, fewer layers		Less dense epithelium		

−, lack of AhR expression; +/−, weak positive reaction, single cells; +, positive reaction; ++, strong positive reaction; Control, Group 1; DMSO, Group 2; TCDD, Group 3; vit. E, Group 4; LVM, Group 5; ASA, Group 6; ASA + vit. E, Group 7

The hearts of the control group (Group 1) embryos had different levels of AhR expression in different locations. Weak positive reactions were found in the atrial muscle cells, but negative reactions were found in the ventricular cells. The walls of the atria and ventricles showed different levels of myocyte and blood vessel development.

The hearts of the control group with DMSO-added (Group 2) embryos had myocyte hypertrophy, the myocytes being clearly thicker and more compact than in the control group. The vascular bed was decreased compared with the control group. Weak positive reactions were found in the ventricles and atria, but a negative reaction was found in the apex of the heart.

The embryos in the group injected with TCDD (Group 3) showed very strong AhR expression throughout the heart (Fig. 2), but some parts of the apex of the heart showed weak positive or negative reactions. The muscle cells showed hypertrophy, and the vascular bed was decreased compared with the control group.

The embryos in the group injected which TCDD and vitamin E (Group 4) showed strong positive reactions throughout the heart. Excessive adipose tissue was present, and AhR expression was found in this tissue.

Positive reactions were also found in the embryos in the group treated with TCDD and levamisole (Group 5), but the reactions were weaker than in Groups 3 and 4. AhR expression was found in the atrium, myocardium, and adipose tissue cells. Positive reactions were also found at the apex of the heart.

Only weak AhR expression was found in the hearts of the embryos in the group injected with TCDD and ASA (Group 6), as is shown in Fig. 2. Positive reactions were found in the ventricular myocytes, but a negative reaction was found in the atrium.

The embryos in the group injected with TCDD, ASA, and vitamin E (Group 7) showed AhR expression throughout the heart, but the reaction was less intense than in Group 3 (Fig. 2). The visible reaction was weaker in the atrium than in the ventricles. The heart was less developed than in the control group (i.e., hypertrophy of myocytes was not found).

The results found for the liver samples are shown in Table 2 and Fig. 3. The livers of the control group (Group 1) embryos collected on day 13 were at different stages of development. Large sinusoidal-type vessels were found in many areas, and they formed a network containing blood cells. The accompanying hepatocytes were organized into the first primitive lobules. Locally very weak positive AhR reactions were found in the hepatocytes. The hepatocytes were strongly developed in other areas, resulting in the lumen of the sinusoidal vessels narrowing, giving the appearance of an adult liver.

The embryos in the group with DMSO added (Group 2) were less developed than the control group embryos. Single cells exhibited very weak AhR expression, but negative reactions were mainly found.

Similar hepatic development was observed in the embryos in the group injected with TCDD (Group 3) as in Group 2. Stronger AhR expression was found in the hepatocytes than was found in the other groups, but the positive reactions were not uniformly dispersed over the surface of the liver (Fig. 3). Fat cells were locally prevalent and were visible between the hepatocytes.

The livers from the embryos in the group treated with TCDD and vitamin E (Group 4) had areas in which less morphological differentiation had occurred than expected, and AhR was expressed very weakly in individual cells in these areas. Fat droplets and lymphocytic infiltrations were found in areas between cells. Hepatocytes in these areas showed regressive changes and positive reactions (Fig. 3).

The embryos in the group injected with TCDD and levamisole (Group 5) had well-developed livers similar to mature livers. Positive AhR expression was found in some areas in the liver, but the intensity was weaker than in Group 3.

Single hepatocytes showed AhR expression in the embryos in the group injected with TCDD and ASA (Group 6). Steatosis in the hepatocytes was observed (Fig. 3), and the lobe structure of the liver was locally obliterated.

The livers of the embryos in the group injected with TCDD, ASA, and vitamin E (Group 7) were less differentiated than the control group livers. Single cells exhibited AhR expression. Steatosis within the hepatocytes was found locally.

The brain tissue results for the samples collected on day 13 are shown in Table 2. The correct brain structure was beginning to form in the embryos in the control group (Group 1). The developing brain contained five layers. Weak positive reactions were observed in areas with migrating and proliferating cells and in the ependyma cells. Little AhR expression was found in the white matter, but negative reactions were found in the gray matter. Single microglial cells in the mesenchyme showed positive reactions.

Spots of AhR expression were found in the white matter in the embryos in the control group injected with DMSO (Group 2). Weak positive responses were found in individual cells and in the ependyma and ganglion cells.

The outer gray matter layer was less developed in the group injected with TCDD (Group 3) than in the control group. Intense AhR expression was found on the brain surface. Positive reactions were found in the white matter, and positive reactions were found in the gray matter in the nerve fiber layer in the internal cerebral cortex. Weak AhR expression was found in single mesenchymal cells, but no AhR expression was found in the ganglion cells.

The outer gray matter layer was less developed in the group injected with TCDD and vitamin E (Group 4) than in the control group. AhR expression was found in single cells between the cerebral cortex layers and in the internal granular layer. Weak positive reactions were found in the white matter and in the surface layer of the cerebellum.

A strongly developed future internal pyramidal cell layer was found in the group injected with TCDD and levamisole (Group 5). AhR expression was visible in single cells in the outer layer and the internal granular layer of the gray matter. Strong positive reactions were found in the mesenchyme, midbrain, and white matter.

No abnormalities were found in the brains of the embryos in the group injected with TCDD and ASA (Group 6), but the outer gray matter layer was less developed than in the control group. Positive reactions were found only in the white matter cells, and AhR expression was not found elsewhere in the brain.

The visible gray matter layer was strongly developed in the group treated with TCDD, ASA, and vitamin E (Group 7), but the brain was less dense than in the other groups. AhR expression was found in the neurocytes organized into the nucleus of the white matter and in the cerebral cortex, but negative reactions were found elsewhere in the brain.

The results for the eye tissues are shown in Table 2. The eye epithelium was highly evolved, and no AhR expression was found in the control group (Group 1). The eye epithelium in the group treated with DMSO (Group 2) contained seven layers, and weak positive reactions were found in the surface layer.

Developmental disorders were found in the six-layer eye epithelium found in the group injected with TCDD (Group 3). Weak positive reactions were found in the external granular layer of the epithelium.

Eye epithelium development was normal in the group injected with TCDD and vitamin E (Group 4), and no AhR expression was found. The eye epithelium was poorly developed and had a low cell density in the group treated with TCDD and levamisole (Group 5), and AhR expression was not found. Eye epithelium development was normal in the group injected with TCDD and ASA (Group 6), and weak positive reactions were found on the surface of the epithelium at the retina. The eyes of the group treated with TCDD, ASA, and vitamin E (Group 7) were similar to the eyes in Group 6, the eye epithelium being properly developed. However, the surface layer was thick and a low level of AhR expression was found.

### Effects of TCDD on COX-2 expression

#### Seven-day-old chicken embryos

**Fig. 4:**
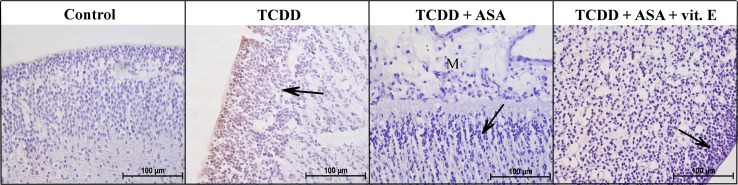
COX-2 expression in the brain of the selected groups of 7-day-old chicken embryos (magnification ×400). Control—Group 1, TCDD—Group 3, TCDD + ASA—Group 6, TCDD + ASA + vit. E—Group 7. Developmental disorders of cerebral cortex (*arrows*) associated with retardation in cell proliferation (Group 7), migration and meninges organization (M) (Group 6), and COX-2 expression and cell adhesion (Group 3)

The COX-2 expression results are shown in Table 3 and Fig. 4.

**Table 3:** Expression of COX-2 in different tissues/organs and groups of 7-day-old chicken embryos

Organ\group	Control	DMSO	TCDD	Vit. E	LVM	ASA	ASA + vit. E
Epidermis	−	−	−	−	−	−	−
Eye epithelium	−	−	−	−	−	−	−
Mesenchyme	−	−	+	+/−	−	−	−
Brain	−	−	+/−	−	−	−	−
Cartilage with perichondrium	−	+/−	+	+/−	−	−	−
Liver	+/−	−	+/−	+/−	+/−	−	−
Kidneys	+/−	+/−	+/−	+/−	+/−	+/−	+/−

−, lack of COX-2 expression; +/−, weak positive reaction, single cells; +, positive reaction; Control, Group 1; DMSO, Group 2; TCDD, Group 3; vit. E, Group 4; LVM, Group 5; ASA, Group 6; ASA + vit. E, Group 7

No COX-2 expression was found in any tissue in the control group (Group 1) embryos on day 7, but some liver and kidney samples had weak positive reactions. Weak positive reactions were observed in the kidney and locally in the cartilage in the embryos in Group 2 (which had been treated with DMSO). No COX-2 expression was found in other tissues such as the epidermis, eye epithelium, brain, and liver. No COX-2 expression was found in the mesenchyme.

Positive reactions were found in the ependyma cells of the brain (Fig. 4) and the mesenchyme below the eye epithelium in the group injected with TCDD (Group 3). COX-2 expression was also found in the cartilage matrix in the bone primordia. Weak and sporadic positive reactions were found in the liver and kidneys.

Embryos in the group treated with TCDD and vitamin E (Group 4) showed weak positive reactions in the mesenchyme, bone primordia cartilage, intervertebral ganglia, liver, and proximal tubules in the kidney. Only a few embryos in the group injected with TCDD and levamisole (Group 5) showed weak positive reactions in the liver and kidneys. Embryos in the group injected with TCDD and ASA (Group 6) showed no COX-2 expression in most of the tissues. Weak COX-2 expression was found in the ganglion cells and proximal tubule cells in the kidneys. Embryos in the group treated with TCDD, ASA, and vitamin E (Group 7) showed weak positive reactions only in the proximal tubules of the nephron.

#### Thirteen-day-old chicken embryos

The COX-2 expression results are shown in Table 4.

**Table 4:** Expression of COX-2 in different tissues/organs and groups of 13-day-old chicken embryos

Organ\group	Control	DMSO	TCDD	Vit. E	LVM	ASA	ASA + vit. E
Heart							
Ventricle	−	−	−	−	−	−	−
Atrium	+/−	−	+/−	+/−	+/−	+/−	+/−
Apex	−	−	−	−	−	−	−
Developmental disorders		Myocyte atrophy, steatosis	Steatosis		Single steatosis		
Liver							
Reaction	−	−	+/−	−	+/−	+/−	+/−
Developmental disorders			Steatosis, lymphocytic infiltrations	Steatosis, lymphocytic infiltrations, areas of necrosis	Single steatosis	Blurred organ structure	Single steatosis
Brain	−	−	−	−	−	−	−
Eye	−	−	−	−	−	−	−

−, lack of COX-2 expression; +/−, weak positive reaction, single cells; +, positive reaction; Control, Group 1; DMSO, Group 2; TCDD, Group 3; vit. E, Group 4; LVM, Group 5; ASA, Group 6; ASA + vit. E, Group 7

The atrial muscle cells of the hearts of the control group (Group 1) embryos collected on day 13 expressed COX-2 weakly, but negative reactions were found in the ventricular myocardium. COX-2 was not expressed throughout the heart in the group treated with DMSO (Group 2), but myocyte atrophy and steatosis were found.

A weak positive response was found in the heart atrium in the group injected with TCDD (Group 3), but the ventricles and apex did not express COX-2. Fat cells were found between the myocytes.

Mainly negative reactions were found in the group injected with TCDD and vitamin E (Group 4). Weak expression of COX-2 was only found in the cardiac muscle cells. Slight positive reactions were found in individual cells in the embryos in the group injected with TCDD and levamisole (Group 5), but no COX-2 expression was found in most of the heart, although sparse steatosis was found between myocytes. A similar COX-2 expression pattern was found in the group treated with TCDD and ASA (Group 6), with weak positive reactions only in the atrium cells but negative responses being found in the ventricles. Similarly, weak positive reactions were only found in the atrium in the group injected with TCDD, ASA, and vitamin E (Group 7), and no COX-2 expression was found in the rest of the heart.

No COX-2 expression was found in the livers of the control group (Group 1) embryos or the embryos in the group treated with DMSO (Group 2), as is shown in Table 4. The reverse was found for the group injected with TCDD (Group 3), with COX-2 being found in single hepatocytes. Lymphocytic infiltrates were also found in the liver.

Numerous lymphocytic infiltrations and necrotic liver cells were found in the group treated with TCDD and vitamin E (Group 4). Vacuolization was found in many of these cells, indicating that the hepatocytes had accumulated fat. The characteristic structure of the liver also became amorphous and no COX-2 expression was found. The livers in the group injected with TCDD and levamisole (Group 5) showed positive reactions and sparse steatosis. Blurring of the structures and membranes between the hepatocytes and weak COX-2 expression were found in the livers from the group treated with TCDD and ASA (Group 6). Fatty liver cells and weak positive reactions were found in the group injected with TCDD, ASA, and vitamin E (Group 7).

No COX-2 expression was found in the brains of the control group or the group treated with DMSO (Table 4). No COX-2 expression was found in any of the brains from the embryos treated with TCDD or TCDD and vitamin E, levamisole, ASA, or ASA plus vitamin E.

No COX-2 expression was found in the eyes of the control group embryos or the embryos treated with DMSO (Table 4). No positive staining in any of the eye was found in the embryos treated with TCDD or with TCDD and vitamin E, levamisole, ASA, or ASA plus vitamin E.

## Discussion

Dioxins are insidious poisons because they are organoleptically undetectable and are toxic even at low concentrations. TCDD at a concentration of 5 µg/kg b.w. has toxic effects that manifest themselves even in the second generation of experimental animals (Całkosiński 2005). For comparison, the toxic gas nitrogranulogen is used as a cytostatic at a concentration of 600 µg/kg body weight (b.w.) but has immunomodulatory properties at a concentration of 5 µg/kg b.w. Dioxins affect the body in multiple ways, causing reproduction and development disorders, immunotoxicity, thymus involution, liver damage, and cancer. Moreover, dioxin exposure results in the formation of reactive oxygen species that cause oxidative stress in cells and tissues and stimulate inflammation. This can lead to DNA damage, decreased mitochondrial respiration, and decreased NAD^+^ concentrations (Całkosiński 2008; Yoon et al. 2006).

It has been found in numerous studies that the actions of dioxins at the molecular level are connected to cytosolic AhR, which activates the transcription of many genes, particularly xenobiotic metabolizing enzymes, including cytochrome P-450 (Brauze et al. 2006). In an experiment on chicken embryos performed by Walker et al. (2000), the AhR protein was found in nerve ganglia, the heart, skeletal and smooth muscle, epithelial tissues, mesenchyme tissues, the kidneys, and the liver.

Fernandez-Salguero et al. (1996) proved that the AhR plays an important role in the mechanism through which dioxins act. They showed that *AhR* ^−^ transgenic mice exposed to TCDD at a dose of 2000 µg/kg b.w. did not suffer damage to the heart, thymus, liver, kidney, pancreas, spleen, or lymph nodes.

In this study, we found that exposure to TCDD caused the AhR to be expressed in the early stages of embryo development (on day 7) in the skin, eye epithelium, liver, skeletal cartilage primordia, kidneys, brain, and mesenchymal cells. The expression of the AhR in the dorsal skin and mesenchymal cells was unchanged at the same time in the control group, suggesting that the AhR is involved in developmental processes. In previous publications, it has been indicated that the AhR plays key roles in the proliferation and differentiation of cells including T lymphocytes (Quintana et al. 2008), neurons (Akahoshi et al. 2006), hepatocytes (Walisser et al. 2005), and cells formed during hematopoiesis (Gasiewicz et al. 2010; Tijet et al. 2006).

Całkosiński (2008) exposed rats to TCDD and treated the rats for 3 weeks with high tocopherol doses (30 mg/(kg b.w. days)). They found that tocopherol effectively decreased the concentrations of proinflammatory cytokines in the serum, limiting inflammation and the damage caused by TCDD. The antioxidant properties of vitamin E have also been demonstrated in mice exposed to TCDD. Injecting tocopherol in a single dose of 150 mg/kg b.w. and then in doses of 40 mg/kg b.w. on five consecutive days significantly decreased the symptoms of TCDD poisoning. Treatment with tocopherol has been found to decrease the concentration of nitrogen peroxide and decrease the incidence of damage to single-stranded DNA (Alsharif and Hassoun 2004). Kloser et al. (2011) demonstrated that tocopherol is antagonistic to TCDD and prevents TCDD from interacting with the AhR.

In a recent search for compounds that decrease the negative effects of dioxins, ASA was identified as a pharmacological agent that has anti-inflammatory and antioxidant properties and that acts as an AhR antagonist. In studies using rats and human hepatoma cells, it has been found that ASA can block the AhR, preventing dioxins being transformed within the body and having negative effects (MacDonald et al. 2004). ASA also inhibits both COX-1 and COX-2, contributing to them being acetylated (Czyż and Watała 2005). In our study, vitamin E was used at a dose of 30 µg/g egg, and its effect was boosted by the presence of ASA at a dose of 50 µg/g egg. The effects of ASA occur through changes in AhR and COX-2 expression.

Dioxins affect the immune system, so we explored the potential protective effects of levamisole, a known immunomodulator that influences both primary and secondary immune responses.

Administering vitamin E decreased AhR expression in the organs except the dorsal epidermis, brain, and mesenchyme of the embryos collected on day 7. Levamisole inhibited AhR expression in all of the organs and simultaneously halted the development of the embryos collected on day 7. The most important changes in the embryos treated with levamisole compared with the control embryos were weaker cell migration in the brain and an underdeveloped ectodermal epithelium. The embryos treated with ASA or ASA and vitamin E expressed the AhR only in the kidney primordia. The gray matter in the brain was also less developed in the embryos treated with ASA and vitamin E than in the control embryos.

It has been confirmed in numerous studies that dioxins are cardiotoxic and hepatotoxic. It has been shown in experiments using chicken embryos that TCDD causes structural changes in the heart by causing cardiomyocyte proliferation disorders (Ivnitski et al. 2001; Walker and Catron 2000; Walker et al. 1997). The structural changes include interventricular septum defects, cardiac hypertrophy, and extension of the ventricle cavity, which is associated with the heart walls being thinner than in unaffected embryos. Hilscherova et al. (2003) found that dioxins cause oxidative DNA damage in chicken embryo livers by increasing the amounts of reactive oxygen species produced and depleting glutathione.

In our study, TCDD caused adverse changes in the heart, liver, brain, and eyes of the embryos collected on day 13. Myocardial hypertrophy, delayed liver development, and steatosis in numerous organs were found. The gray matter in the brain was also less developed in the embryos exposed to TCDD than in the control embryos, and developmental disorders of the eye epithelium were found in the embryos exposed to TCDD. AhR expression was found in all of the organs and groups, but the most intense AhR expression was found in the group injected with only TCDD. ASA was the most effective of the protective substances used. ASA decreased AhR expression and the negative effects of TCDD on embryo development, especially the development of the heart, liver, and eyes. Similar results were found for the group treated with ASA and vitamin E, although the protective effects on the heart appeared to be slightly weaker than the effects of ASA alone. Levamisole had protective effects on organ development and decreased AhR expression. Vitamin E had no protective effects on organ development in the embryos collected on day 13, and the organs from this group had similar developmental levels to the organs from the group injected with only TCDD, but slightly less AhR expression was found in the eyes and brain.

In previous studies, it has been found that exposure to dioxins leads to inflammation. TCDD causes elevated levels of the proinflammatory cytokines interleukin-1, interleukin-6, and tumor necrosis factor and therefore increases COX-2 activity. This leads to proinflammatory prostaglandins and thromboxanes being synthesized and the inflammation of tissues (Nishimura et al. 2008).

Weak COX-2 expression was found in the liver and kidneys of the embryos collected on day 7. TCDD also induced COX-2 expression in the mesenchyme cells, brain, and cartilage and perichondrium. The protective substances except vitamin E inhibited COX-2 expression in all of the organs except the kidneys, but levamisole did not inhibit COX-2 expression in the liver.

The embryos collected on day 13 showed COX-2 expression in the heart atrium and liver. COX-2 expression was not found in the brain or eyes of the embryos in any group. The protective substances did not inhibit COX-2 expression in the embryos collected on day 13. Lipocytes were observed within the myocardium in the embryos exposed to TCDD, and this may affect the future functioning of the heart. Adding vitamin E, ASA, or ASA plus vitamin E suppressed the formation of fat cells between the myocytes. COX-2 expression in the liver was associated with steatosis occurring between the hepatocytes and lymphocytic infiltration and inflammation in the embryos treated with TCDD and with TCDD and vitamin E. Lymphocytic infiltrates were not observed in the embryos in the other groups even though COX-2 expression and visible lipocytes were found. These results indicate that ASA, levamisole, and ASA plus vitamin E have effective immunomodulatory activities and can decrease the damage dioxins cause to organs.

Our results support the results of previous studies in which dioxins were found to affect AhR and COX-2 expression in chick embryo tissues (Fujisawa et al. 2014; Karchner et al. 2006; Walker et al. 1997). We successfully demonstrated that vitamin E, levamisole, and ASA modulate the negative effects of TCDD. The protective compounds inhibited AhR and COX-2 expression in the embryos collected on day 7 but did not have strong protective effects on the embryos collected on day 13. This may be because the injected compounds were metabolized as the embryo developed and because the mass of the embryo increased between days 7 and 13, weakening the pharmacological effects of the test compounds. However, new receptors could be created during embryogenesis that reconstitute and develop with organogenesis. This may be why some positive reactions were found in the embryos collected on day 13. ASA, levamisole, and ASA plus vitamin E weakened the immune response to TCDD and prevented changes in multiple organs. Vitamin E did not fully protect against developmental changes. The main conclusions of the study are summarized below.

- TCDD increased AhR and COX-2 expression in the tissues of chicken embryos collected on days 7 and 13 of incubation.
- TCDD caused developmental abnormalities in the chicken embryos, especially in the heart, liver, brain, and eyes.
- Vitamin E, levamisole, ASA, and ASA plus vitamin E inhibited AhR and COX-2 expression in the tissues of embryos exposed to TCDD collected on day 7 of incubation and decreased AhR and COX-2 expression in the tissues of embryos collected on day 13.
- ASA, levamisole, and ASA plus vitamin E weakened the immune response and prevented multiple organ changes. However, vitamin E did not completely protect against changes in the developing embryos.
